# Nano‐Engineered Titanium Implants Loaded With Gingival Fibroblasts‐Derived Microvesicles Enhance Early Osseointegration And Soft Tissue Attachment In Vivo

**DOI:** 10.1002/adhm.202504516

**Published:** 2026-01-15

**Authors:** Pingping Han, Chun Liu, Anjana Jayasree, Kamil Sokolowski, Andrew Liaw, Dingbin Liu, Carlos Salomon, Karan Gulati, Ryan SB Lee, Baoxin Huang, Sašo Ivanovski

**Affiliations:** ^1^ School of Dentistry The University of Queensland Herston Queensland Australia; ^2^ School of Dentistry Centre For Orofacial Regeneration, Reconstruction and Rehabilitation (COR3) The University of Queensland The University of Queensland Herston Queensland Australia; ^3^ UQ Centre for Extracellular Vesicle Nanomedicine The University of Queensland Brisbane Queensland Australia; ^4^ School of Pharmacy and Pharmaceutical Sciences The University of Queensland Woolloongabba Queensland Australia; ^5^ Translational Research Institute Woolloongabba Queensland Australia; ^6^ State Key Laboratory of Medicinal Chemical Biology Research Center For Analytical Sciences and Tianjin Key Laboratory of Molecular Recognition and Biosensing College of Chemistry Nankai University Tianjin China; ^7^ Faculty of Medicine Centre For Clinical Diagnostics Translational Extracellular Vesicles in Obstetrics and Gynae‐Oncology Group University of Queensland Centre For Clinical Research Royal Brisbane and Women's Hospital The University of Queensland Brisbane Queensland Australia; ^8^ Guangdong Provincial Key Laboratory of Stomatology and Guanghua School of Stomatology Hospital of Stomatology Sun Yat‐sen University Guangzhou China

**Keywords:** extracellular vesicles, in vivo osseointegration, in vivo soft tissue healing, titania nanopores

## Abstract

Titanium dental implants require both reliable osseointegration and peri‐implant soft tissue seal formation to ensure long‐term success. While osseointegration has been widely studied, strategies to optimize soft tissue healing remain underexplored. In this study, we developed titania nanopore (TNP) implant surfaces functionalized with human primary gingival fibroblasts‐derived microvesicles (hGFs‐MVs) to investigate early peri‐implant soft and hard tissue healing in vivo. hGFs‐MVs were enriched, characterized, and loaded onto TNP implants at 10^9^ particles per implant (2 mm × 3 mm) via passive adsorptive loading. Rat first molars were extracted, and implants were placed into healed sockets after 4 weeks. Animals were assigned to TNP or hGFs‐MVs/TNP groups (n = 5–6) and assessed at 1 and 4 weeks by micro‐CT and histology. Cryo‐electron microscopy confirmed the double‐membrane structure of hGFs‐MVs, and in vitro release studies showed peak vesicle release at day 3. Histology revealed early osteoid formation at week 1 and mature mineralized bone by week 4. hGFs‐MVs/TNP implants exhibited significantly higher percentage of bone‐to‐implant contact (BIC%) at 4 weeks. Importantly, the hGFs‐MVs/TNP group showed markedly enhanced soft tissue attachment and mature collagen fibers. These ‘proof‐of‐concept’ findings suggest that hGFs‐MVs‐loaded TNP implants enhance both in vivo osseointegration and soft tissue integration, providing a clinically relevant, cell‐free strategy for improving peri‐implant healing.

## Graphical abstract

Our study showed that human primary gingival fibroblast–derived microvesicles on nano‐engineered titanium implants promote early osseointegration and soft‐tissue attachment in vivo.

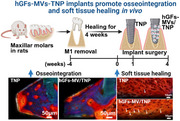

## 1 Introduction

The establishment of a robust soft tissue seal in conjunction with osseointegration is essential for the long‐term success of titanium dental implants [1, 2], due to their transmucosal nature that exposes them to a challenging microbial‐rich oral environment [3]. Often, the complications of suboptimal peri‐implant soft tissue health and/or bone loss begin with an inadequate soft tissue seal, which predisposes bacterial ingress, inflammation, and propagation of peri‐implant disease [1, 2, 4]. Consequently, there has been increasing interest in optimizing implant surface characteristics, especially micro and macrodesigns, for a more resilient soft tissue attachment [2].

Nanoscale topographical modification, such as titanium nanotubes (TNTs) and nanopores (TNPs), mimics the extracellular matrix and can influence connective tissue cell adhesion, proliferation, and differentiation [5, 6, 7, 8]. Among these, TNPs offer a scalable and controllable approach for bioactive nanostructure design and have shown promise in promoting in vivo osseointegration [9, 10, 11]. However, their synergistic potential with extracellular vesicles in enhancing soft tissue healing has yet to be fully elucidated.

Extracellular vesicles (EVs) are membrane‐bound nanoparticles secreted by virtually all cell types and are broadly classified into three subtypes based on their biogenesis: apoptotic bodies, microvesicles (MVs), and exosomes (small EVs, typically <200 nm) [12, 13, 14]. Large EVs, including apoptotic bodies and microvesicles (100–1000 nm), are formed by outward budding of the plasma membrane, while exosomes originate from endosomal membranes and are released upon fusion of multivesicular bodies with the plasma membrane [15]. Microvesicles carry membrane proteins, cytosolic components, and bioactive molecules (ie, DNA/RNA, cytokines) from their parental cells, enabling them to exert potent paracrine effects in intercellular communication, wound healing, tissue regeneration, and immune modulation [16, 17]. For example, gingival epithelial‐derived MVs can promote inflammation and mineralization in gingival fibroblasts [17, 18], whereas MVs from human primary gingival fibroblasts (hGFs‐MVs) have been shown to promote angiogenesis, whereas hGFs‐exosomes enhance osteogenesis in vitro [19]. Because of their functional diversity, EVs have been increasingly investigated as therapeutic agents for modifying titanium implant surfaces. Recent studies have employed TNTs or TNPs as delivery vehicles for exosomes to enhance in vivo bone integration in tibia or skull defects (reviewed in [6]). Specifically, TNF‐α‐treated mesenchymal stem cell (MSC)‐exosomes [20], human dental pulp stem cells‐exosomes [21], and M2 macrophage‐exosomes (M2‐sEVs) [22] have been immobilized on TNTs or TNPs surfaces, which enhanced osseointegration in vivo. However, their application in the context of osseointegration and soft tissue healing in dental implants in vivo, particularly in combination with human gingival fibroblast‐derived microvesicles (hGFs‐MVs), is yet to be elucidated. Given the proven role of gingival fibroblasts (GFs) as a critical cell population to modulate local tissue responses by influencing connective tissue behavior and contributing to extracellular matrix remodeling at the implant interface subsequent to dental implantation [23, 24], their extracellular vesicles may contain key bioactive molecules from the parental cells, enabling them to exert similar regulatory effects. Initial studies evaluating hGFs‐MVs‐releasing TNTs demonstrated a reduced epithelial inflammation in vitro [25]. Therefore, we hypothesized that TNP surfaces functionalized with hGFs‐MVs (via passive adsorptive loading) would enhance fibroblast adhesion and collagen maturation in early healing, thereby promoting a stable soft tissue interface while maintaining favorable conditions for bone integration. This study aimed to investigate the in vivo effects of hGFs‐MVs‐loaded TNP implants in enhancing early peri‐implant soft tissue integration, without compromising osseointegration.

## 2 Materials and Methods

### 2.1 Experimental Design

**FIGURE 1:**
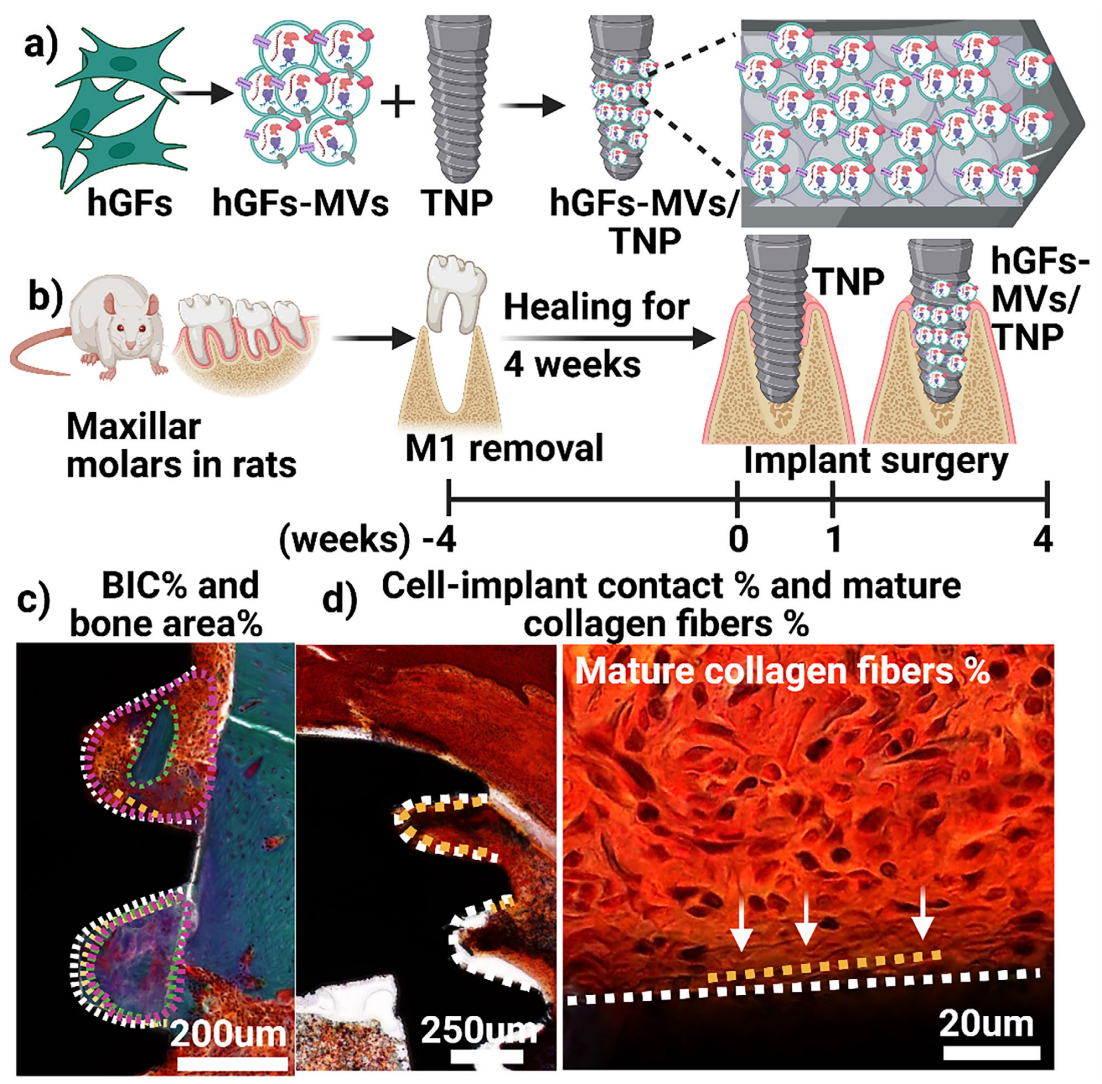
Workflow for experimental design. (a) Microvesicles derived from human primary gingival fibroblasts (hGFs‐MVs) were isolated and subsequently loaded onto nanoengineered titanium implants with titania nanopores (TNP) surfaces. (b) In vivo surgical model: the maxillary first molar (M1) was extracted, followed by a 4‐week healing period. hGFs‐MVs‐TNT implants were then placed at the M1 site and evaluated at 1‐ and 4‐week post‐implantation. (c) Osseointegration was calculated by bone‐to‐implant contact (BIC %)—percentage of yellow line (bone contact) length to white line (total implant) length, and bone area (%)—Ratio of bone area (green circled) to total implant area (purple circled). (d) Peri‐implant soft tissue quantification was assessed by peri‐implant connective tissue cell to implant contact (%)—the percentage of yellow line (connective tissue cell attachment to implant surface) length to white line (total implant) length and mature collagen fibers (%)—the ratio of the yellow line (mature collagen fiber length) to the white line (total implant length). White arrows indicate mature collagen fibers.

In this study, microvesicles were isolated from human primary gingival fibroblasts (hGFs‐MVs) using centrifugation at 16 000 g, and subsequently loaded onto nano‐engineered titanium implants with nanopores (TNP) via passive adorptive loading (Figure 1a). Bilateral extraction of the rats' maxillary first molars was performed, followed by a 4‐week healing period before implantation of either TNP alone or hGFs‐MVs‐loaded TNP implants into the extraction sockets. Samples were collected at 1‐ and 4‐week post‐implantation for micro‐CT and resin sectioning for histological analyses (Figure 1b).

### 2.2 Fabrication of TNPs on Titanium Implants by Electrochemical Anodization

Moderately rough titanium implants (2 mm diameter, 3 mm length) were custom‐manufactured by Southern Implants Ltd (Irene, South Africa) using Type IV commercially pure titanium. Before anodization, Ti implants were cleaned thoroughly by sonication in ethanol twice and finally air‐dried. An appropriately aged ethylene glycol (≥ 99 %; Sigma–Aldrich, Australia) electrolyte comprising 5 % water (v/v) and 0.3 % Ammonium fluoride (NH_4_F, ≥ 99 %; Sigma–Aldrich, Australia) was used for anodization [26]. Before anodizing the target substrate, the electrolyte was pre‐conditioned (aged) by repeated anodization of a non‐target Ti substrate, as described previously [27, 28]. Ti implants (anode) and a non‐target Ti implant (cathode) were immersed in the electrolyte, followed by anodization at 80 V for 60 min using Keysight E36106A DC power supply to fabricate nanopores (TNPs). TNPs implants were washed in deionized water and dried in air before use.

Anodized titanium (Ti) implants were mounted on 25 mm diameter Ted Pella pin stubs using double‐sided conductive carbon tape (Ted Pella) and coated with a 5 nm platinum layer to enhance surface conductivity. Scanning electron microscopy (SEM) was performed using field emission scanning electron microscopes (FESEM), including a JEOL JSM‐7100F.

### 2.3 Enrichment and Characterization of hGFs‐MVs

Human primary gingival fibroblasts (hGFs) were isolated from abundant dental tissue of a 37‐year‐old female donor following third molar extraction under approved human ethics by The University of Queensland Human Ethics Committee (approval number 2019000134). At 90 % confluency, hGFs were rinsed with Dulbecco's phosphate‐buffered saline (DPBS, no calcium, no magnesium; Thermo Fisher Scientific) and cultured in serum‐free Dulbecco's modified Eagle's medium (DMEM; Gibco‐Invitrogen) with antibiotics Gibco Antibiotic–Antimycotic (Thermo Fisher Scientific) for 16 h to collect conditioned media (CM) prior to MV isolation. hGFs‐MVs were isolated using a differential centrifugation method whereby the collected conditioned medium (∼150 mL) was sequentially centrifuged at 300 and 2600 g (15 min each, 4°C) to remove debris and apoptotic bodies, followed by 16 000 g for 20 min to pellet microvesicles (MVs). MVs were resuspended in 500 µL PBS and stored at −80°C for further use.

Enriched hGFS‐MVs were characterized following the latest international extracellular vesicle research guidelines [29]. Morphology was examined using transmission electron microscopy (TEM) and cryogenic electron microscopy (cryo‐EM), size distribution was assessed via nanoparticle tracking analysis (NTA), protein content was measured using the BCA assay, and EV surface markers were analyzed with a multiplex exosome detection kit.

For TEM analysis, 5 µL of hGFs‐MVs were fixed in 3 % glutaraldehyde (w/v), placed on glow‐discharged Formvar carbon‐coated grids, stained with uranyl‐oxalate (pH 7) for 3 min, and imaged using an FEI Tecan 12‐transmission electron microscope (FEI, Hillsboro, OR). For cryo‐EM, 5 µL of hGFs‐MVs (10^10^/mL) were fixed in 4 % paraformaldehyde, applied to a 200‐mesh grid in a vitrification system, rapidly frozen in ethane (−183°C), and imaged with an FEI Talos 200C high‐resolution TEM at −180°C (Thermo Fisher Scientific).

An in‐house enzyme‐linked immunosorbent assay (ELISA), based on the PeproTech TMB substrate kit (Lonza, Australia), was used to detect the EV surface marker CD9. Plates were coated overnight with 2 µg/mL anti‐human CD9 antibody (HansaBioMed, Lonza), blocked with 1 % BSA for 1 h, and incubated with 2 µg/mL biotin‐conjugated CD9 antibody (HansaBioMed, Lonza) for 1 h at 37°C. Streptavidin‐HRP was then added for 30 min, followed by TMB substrate. The reaction was stopped with 1 M HCl, and absorbance was read at 450 nm. Particle size and concentration of hGFs‐MVs were analyzed using the NanoSight NS500 (NanoSight, UK) equipped with a 488‐nm laser. Five 30 s videos per sample were recorded with NTA 3.1 software (camera level 14, detection threshold 5). Polystyrene beads (100 nm) and PBS served as positive and negative controls, respectively. Data were processed to determine particle mode size and concentration.

Using the MACSPlex Exosome Kit (Miltenyi Biotec), EV surface markers were profiled with a bead‐based multiplex flow cytometry assay. Briefly, 20 µL of hGFs‐MVs were diluted to 60 µL with MACSPlex buffer and incubated with 8 µL of capture beads targeting 39 surface markers. Samples were counterstained with APC‐labeled antibodies against CD9, CD63, and CD81, then analyzed on a BD FACSVerse cytometer. Median fluorescence intensity (MFI) was background‐corrected using mIgG1 controls.

### 2.4 EV Loading to TNP Implants and EV Release Profile

The hGFs‐MVs were loaded onto TNP implants following our previously described protocols by passive loading at room temperature [25]. TNP implants were loaded with 1 × 10^9^ MVs per implant for both the in vitro MV release assay and the in vivo implantation, based on previous studies demonstrating effective modulation of inflammatory responses in vitro [25]. Briefly, TNP implants were immersed in a PBS containing 1 × 10^9^ MVs per implant and incubated for 1 h at room temperature to allow passive adsorption‐based attachment of hGFs‐MVs to the implant surface, after which the implants were gently washed with PBS to remove any unattached MVs. AFM measurements were performed in tapping mode to assess surface roughness parameters after MV loading.

Confirmation of successful EV loading to TNP implants, DiO‐labeled hGFs‐MVs were loaded to TNP implants following our previously described protocol [25]. A lipophilic green fluorescent dye, 3,3’‐Dioctadecyloxacarbocyanine perchlorate (DiO), was incubated with hGFs‐MVs particles at 25 µg/mL for 1 h at room temperature. Excess unbound dye was removed using an Amicon Ultra‐0.5 centrifugal filter unit (10 kDa, Merck Millipore) by centrifuging at 4000 × g for 20 min at 4°C. The DiO‐labeled MVs were then visualized using a Nikon confocal microscope. DiO‐labeled MVs implants were visualized via confocal microscopy at an excitation/emission of 484/501 nm.

Next, MV release from TNP implants was evaluated by incubating the implants in 1 mL of PBS at 37°C, with PBS collected and replaced on days 1, 3, and 7. Released MVs were quantified using the BCA protein assay and CD9 ELISA. Pierce BCA protein assay (Thermo Scientific) was used for MV release from Ti substrates following the manufacturer's instructions. CD9 ELISA was used to quantify CD9^+^ MVs released into PBS using methods described in 2.3. As described in Section 2.3, CD9^+^ EVs were quantified from background‐subtracted A450 values, and these values were compared between groups because no commercial standard was available from the manufacturer (Lonza).

### 2.5 In Vivo Implant Surgery Procedures

Twenty‐four 8‐week‐old Wistar rats (male:female = 1:1) were obtained from the Animal Resources Centre (Canning Vale, WA, Australia). All animal procedures were conducted following the approved protocol from the Animal Ethics Committee of The University of Queensland (Ethics approval number: 2021/AE000524). These rats were randomly assigned to two experimental groups: TNP (n = 6) and hGFs‐MV/TNP (n = 6), with evaluations conducted at two time points (1 and 4 weeks) post‐implantation.

Rat first molars were selected because their size and surrounding alveolar bone allow stable implant placement, and their healing characteristics mirror human post‐extraction remodeling. This well‐established dental implant placement model minimizes anatomical variability and has been validated in our previous studies [30, 31]. Briefly, animals were anesthetized with 3.5 % isoflurane inhalation, followed by subcutaneous administration of pre‐emptive multimodal analgesia (buprenorphine, 0.01–0.05 mg/kg; meloxicam, 1 mg/kg) and prophylactic antibiotics (Kefzol, 20 mg/kg; gentamicin, 5 mg/kg). A ∼2 mm crestal incision was made mesial to the first maxillary molars, followed by a full‐thickness mucoperiosteal flap evaluation to expose the mesial root. Bilateral first molars were extracted using a modified spatula and toothed forceps. Implants were incubated with the MV suspension (1 × 10^9^ MVs per implant) for 1 h at room temperature following the previously described EV loading protocol, prior to implantation. After a 4‐week healing period, TNP and hGFs‐MVs/TNP implants were placed into the mesial extraction sockets. Osteotomies were prepared with a 1.2 mm pilot drill and a 1.8 mm final drill (Southern Implants Ltd., Irene, South Africa) under continuous saline irrigation to avoid thermal damage. TNP and hGFs‐MVs/TNP implants were inserted into the prepared sockets with an insertion torque of approximately 15 N·cm, aligning the 2/3 of the implant shoulder with the bone crest. Wounds were closed with resorbable 5‐0 Vicryl sutures (5‐0 Vicryl, Ethicon, NJ, USA). Postoperative analgesia (buprenorphine, 0.01–0.05 mg/kg; carprofen, 4–5 mg/kg) and antibiotics (enrofloxacin, 2.5 mg/kg) were administered intraperitoneally immediately after surgery and continued daily for three days.

### 2.6 MicroCT Analysis of Osseointegration

At 1‐ and 4‐week post‐implantation, animals were sacrificed, and maxillary specimen blocks surrounding the implants were carefully harvested using a diamond‐tipped circular saw with irrigation. The samples were then immediately fixed in 4 % paraformaldehyde for 24 h at 4°C.

Micro‐CT imaging was conducted using a Skyscan 1272 (Bruker, MA, USA) to evaluate bone formation within the defect. Scanning parameters included 90 kV voltage, 111 µA current, 1700 ms exposure, 21 µm voxel size, 0.6° rotation step, 2‐frame averaging, 4×4 binning, and a 1 mm aluminum filter. Data were reconstructed using CTvox and Data Viewer (Bruker), and analysis was performed with CTan software (Bruker).

Micro‐CT analysis of osseointegration was conducted by defining regions of interest (ROIs) as a 0.15 mm zone extending outward from the implant threads. Within this ROI, the percentage of bone volume (%ROI) was calculated to evaluate new bone formation. The total Volume of Interest (VOI, ∼1.5 mm^3^) was defined as a cylinder volume extending 0.15 mm radially around the implant threads to standardize measurements across samples. Bone density was assessed using grayscale intensity values. Bone surface area (mm^2^) was measured to quantify the bone‐implant interface, and the surface area‐to‐volume ratio was calculated to assess the structural complexity and quality of the newly formed bone surrounding the implant.

### 2.7 Histology and Histomorphometry Analysis of Osseointegration and Peri‐Implant Soft‐Tissue Formation

Maxilla samples were dehydrated through a graded ethanol series and infiltrated with a resin mixture (Methyl Methacrylate/Glycol Methacrylate; Technovit 7200, Heraeus Kulzer, Germany) for hard tissue embedding. After polymerization, resin blocks were initially ground using an EXAKT 400 CS micro‐grinding system (EXAKT Apparatebau, Norderstedt, Germany) to expose the embedded specimens. Blocks were then fixed to glass slides and sectioned to an initial thickness of 150–200 µm using the EXAKT 300 cutting system (Exact Apparatebau, GmbH, Norderstedt, Germany). Longitudinal sections (140–200 µm thick) of the maxilla with implants were prepared in a bucco‐palatal direction, parallel to the long axis of the implant, using the EXAKT cutting system (EXAKT Apparatebau, Norderstedt, Germany). Sections were taken as close as possible to the center of the implant, allowing for two sections per implant. These sections were ground to a final thickness of 20 µm using the EXAKT 400 CS micro grinding system. Staining was performed using both Golden trichrome and H&E. The prepared slides were scanned at ×40 magnification using the Olympus Evident VS120 Slide Scanner. Histomorphometric analysis was carried out using QuPath software by a single‐blinded trained examiner.

Osseointegration was assessed using two primary parameters: Bone‐to‐Implant Contact (BIC %) and Bone Area Percentage, with both measurements excluding the implant threads (Figure 1c). Bone‐to‐Implant Contact (BIC %) was determined by calculating the percentage ratio of the length of bone‐contacting regions (marked by yellow lines) to the total implant length (represented by white lines). Bone Area Percentage was measured as the ratio of bone area within the implant region (outlined in green) to the total implant area (outlined in purple).

For the peri‐implant soft tissue in the coronal third of the implants, excluding threads (Figure 1d), the assessment included soft tissue cell‐to‐implant percentage, calculated as the ratio of the soft tissue attachment length (yellow lines) to the total implant length (white lines), reflecting the extent of soft tissue adherence to the implant surface. Mature collagen fiber percentage was evaluated by comparing the length of mature collagen fibers next to the implant surface (yellow line) to the total implant length (white line). Additionally, cell density was assessed by calculating the number of connective tissue cells per pitch, excluding pitches and threads with epithelial ingrowth.

### 2.8 Statistical Analysis

Data are displayed as the mean ± the standard deviation (SD), and graphs were created using GraphPad Prism 10. Data normality was assessed using the Shapiro–Wilk test, with most datasets found to be parametric unless otherwise noted in each a. Thus, a comparison between two groups (TNP vs. hGFs‐MVs/TNP) was conducted using the Mann–Whitney U test (unpaired, two‐tailed) in Prism 10 (GraphPad Software, San Diego, CA, USA). A p‐value of <0.05 was considered statistically significant.

## 3 Results

### 3.1 EV Characterization of hGFs‐MVs

**FIGURE 2:**
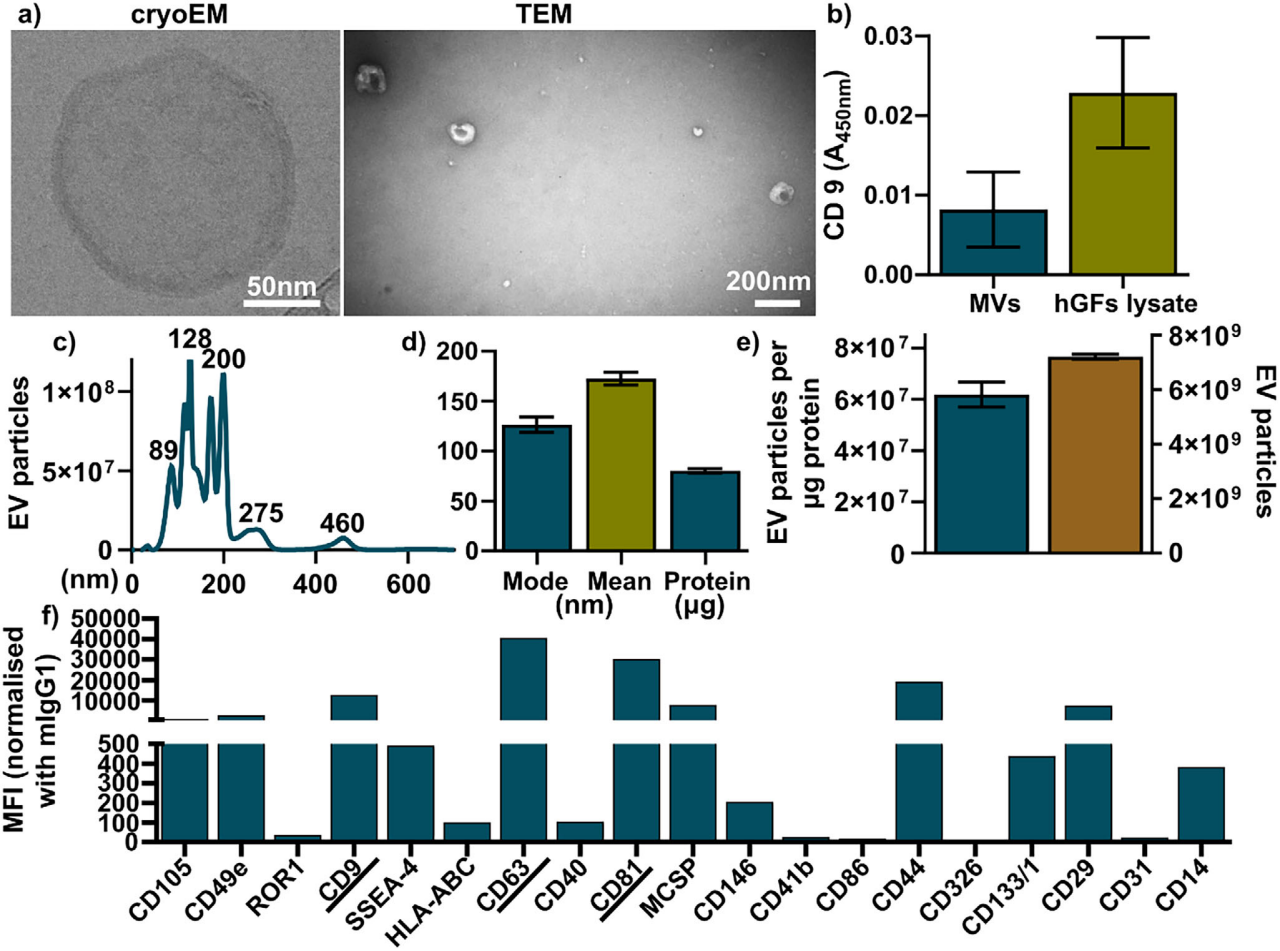
Characterization of hGFs‐MVs. (a) Morphological analysis of hGFs‐MVs using cryo‐electron microscopy (Cryo‐EM) and transmission electron microscopy (TEM). (b) Quantification of the EV surface marker CD9 by ELISA. (c) The size distribution of hGFs‐MVs is shown as a histogram. (d) Summary of EV size (mean and mode) and total MV protein content. (e) EV particle count and purity, expressed as the number of particles per µg of protein. (f) Surface marker profiling using a multiplex EV detection kit, highlighting common EV markers CD9, CD63, and CD81.

Following the latest MISEV guidelines, representative cryo‐EM and TEM images revealed circular EV morphology with a characteristic double‐layered lipid bilayer under cryo‐EM (Figure 2a). CD9 ELISA confirmed the presence of the EV surface marker CD9 on hGFs‐MVs (Figure 2b). Nanoparticle tracking analysis showed that EV particle sizes ranged from 90 to 600 nm, with several peaks observed at approximately 89, 128, 200, 275, and 460 nm (Figure 2c). The mode and mean particle size were both below 200 nm, and total EV protein content was approximately 80 µg (Figure 2d). EV concentration was measured at 7 × 10^9^ particles/mL, with a purity of 6 × 10^7^ particles per µg of protein (Figure 2e). All common EV surface markers—CD9, CD63, and CD81–were detected, with CD63 showing higher expression. Additionally, CD49e, MCSP, CD44, and CD29 were also expressed, each exhibiting MFI values above 10 000.

### 3.2 EV Release From hGFs‐MVs/TNP Implants

**FIGURE 3:**
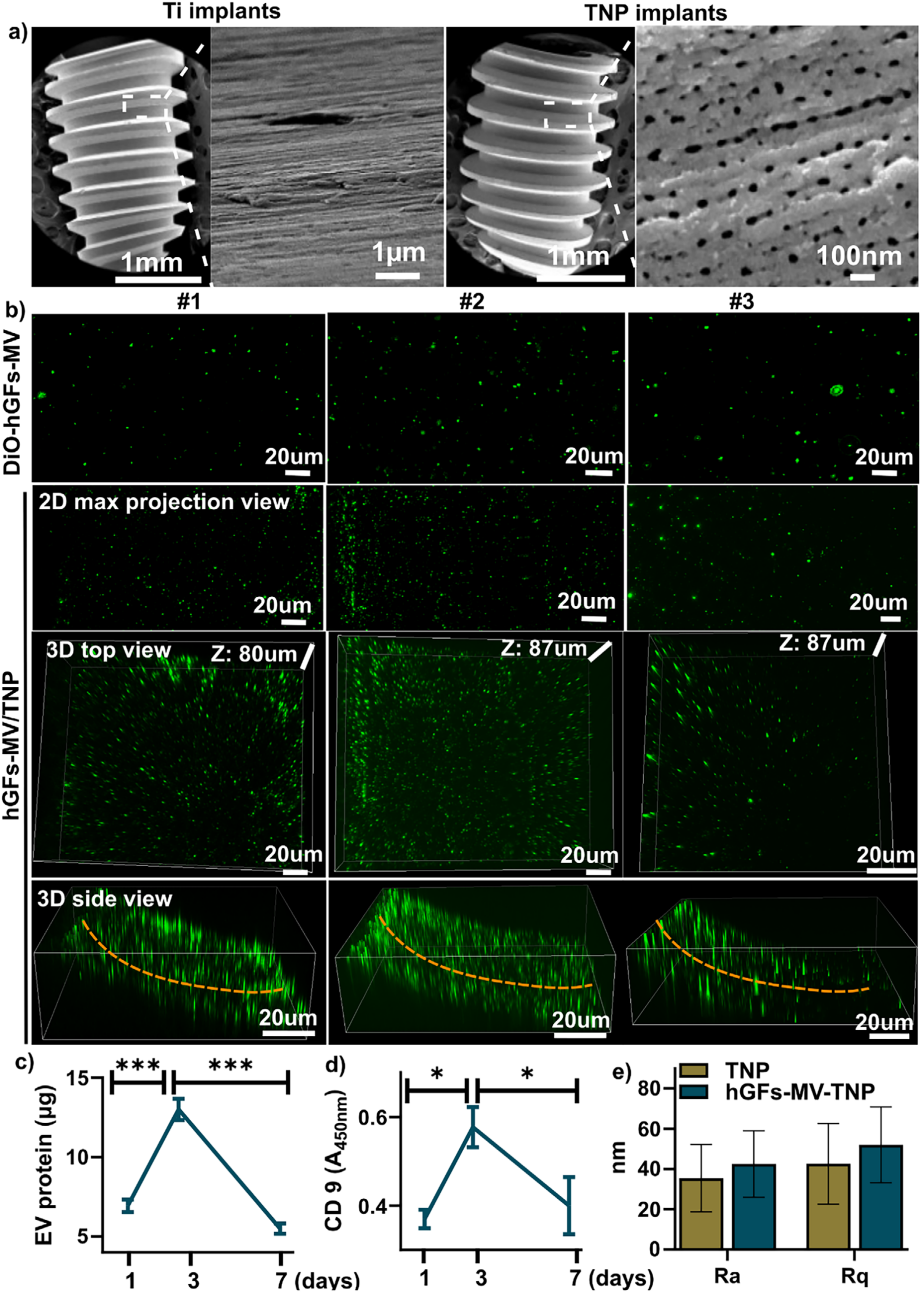
Validation of hGFs‐MVs loading onto TNP implants. (a) SEM images confirmed the successful fabrication of TNPs upon anodization (n = 3). (b) Three representative confocal microscopy images of DiO‐labeled hGF‐MVs (green; top) revealed their distribution on TNP implants, shown as 2D maximum projection images and 3D reconstructions (top and side views), with an overall thickness of ∼80–87 µm (n = 3). #1, #2 and #3 show data from three individual hGFs‐MVs‐TNP implants. (c) EV release was assessed over 7 days using EV protein quantification and CD9 ELISA for hGFs‐MVs‐TNP implants (n = 3). ^*^ *p*<0.05, ^***^ *p*<0.002 between time points.

SEM images of TNP implants revealed a nanoporous surface structure (pore size of 45.8 ± 19.8 nm) following electrochemical anodization (Figure 3a). Three representative images of DiO‐labeled hGFs‐MVs (DiO‐hGFs‐MVs) demonstrated the spherical morphology of the vesicles. Evidence of EV surface loading was visualized using 3D maximum intensity projection, showing green circular DiO‐hGFs‐MVs attached to the TNP implant surface from both top and side views (Figure 3b). BCA assay indicated a peak in EV release at day 3, with significantly higher levels than those at days 0 and 7 (Figure 3c). These findings were further supported by CD9 ELISA, confirming the presence of released EVs (Figure 3d). The AFM roughness results showed that there was no significant difference in surface roughness between the two groups, with TNP implants exhibiting Ra = 42.7 ± 15.9 nm and Rq = 51.3 ± 18.8 nm, while hGFs‐MVs‐TNP samples demonstrated Ra = 42.5 ± 16.6 nm and Rq = 52.1 ± 18.9 nm (Figure 3e).

### 3.3 MicroCT and Histological Analysis of Bone‐Implant Osseointegration

**FIGURE 4:**
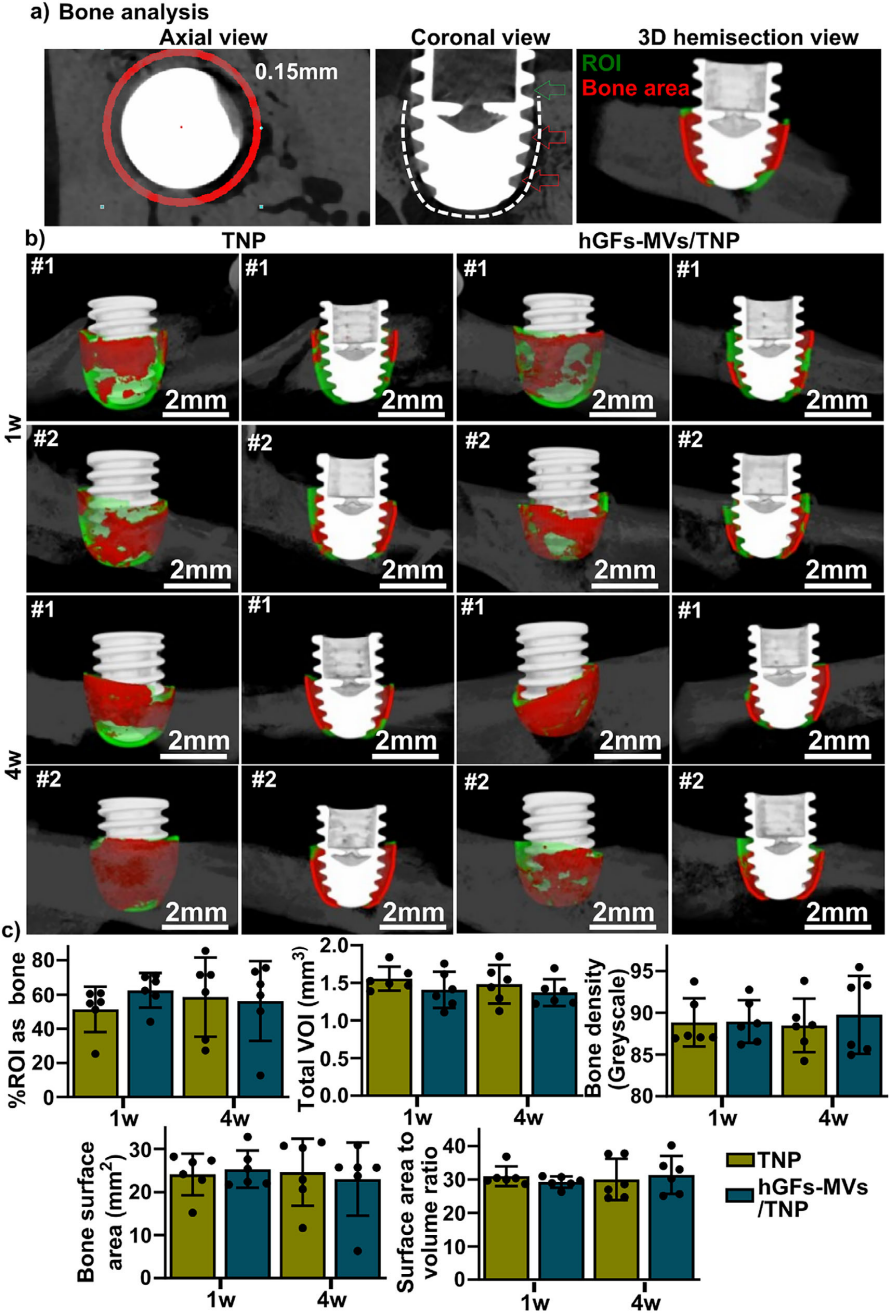
Bone quantification from micro‐CT analysis. (a) A region of interest (ROI) is defined as a 0.15 mm zone in axial (left) and coronal (middle) views surrounding the implant threads in the apical two‐thirds of implants placed subcrustally. A 3D reconstructed hemisection showing segmented bone (red) inside the ROI (green), demonstrating how bone volume was quantified. (b) Two representative reconstructed 3D images showing bone formation within the ROI for both TNP and hGFs‐MVs/TNP implants at 1‐ and 4‐weeks post‐implantation. The right panels showed hemisection views of the corresponding 3D reconstructed images on the left. #1 and #2 indicate data from two individual animals. (c) Quantitative analysis of the percentage of bone within the ROI, total bone volume of interest (VOI, ∼1.5mm^3^), bone density (gray scale values), bone surface area (mm^2^), and surface area‐to‐volume ratio for both TNP and hGFs‐MVs/TNP implants at 1‐ and 4‐weeks post‐implantation (n = 6).

Bone‐implant osseointegration was quantified using microCT images with the ROI set at 0.15 mm surrounding the implants (Figure 4a). Representative reconstructed microCT images of TNP and hGFs‐MVs/TNP implants at 1‐ and 4‐week post‐implantation are shown in Figure 4b. Quantification of microCT data showed no significant differences between the two groups at both time points, with a consistent total volume of interest (VOI) of ∼1.5 mm^3^ for percentage of bone within the ROI, bone density (grayscale values), bone surface area (mm^2^), and surface area‐to‐volume ratio for both implant types at 1 and 4 weeks post‐implantation.

**FIGURE 5:**
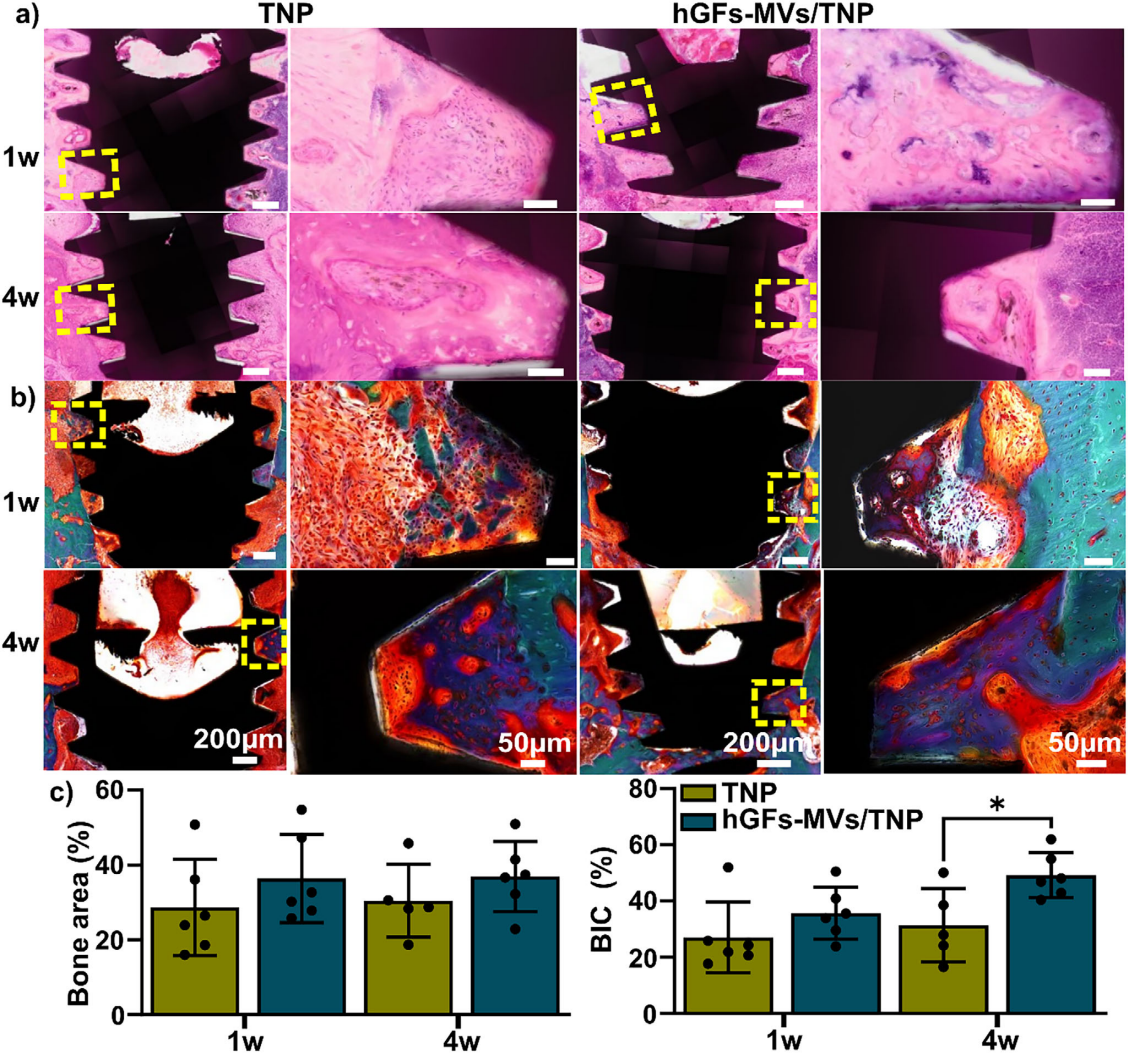
Histological assessment of bone‐implant osseointegration. (a) H&E and (b) Golden trichrome staining of representative histological sections showing bone formation within the implant pitch for TNP and hGFs‐MVs/TNP implants at 1‐ and 4‐weeks post‐implantation. The right panels in (a) and (b) show higher‐magnification views of the yellow‐boxed regions in the corresponding left panels. (c) Quantification of bone area (%) and bone‐to‐implant contact (%)—excluding the implant threads—at 1 and 4 weeks revealed no significant differences between the TNP and hGFs‐MVs/TNP groups (n = 5‐6). ^*^ *p*<0.05 between groups. BIC % at week 1 was analyzed using the non‐parametric Mann–Whitney test, while all other data were analyzed using a two‐tailed unpaired *t*‐test.

Histological images stained with H&E and Golden trichrome, showing implant threads located below the bone level, were presented in Figure 5a,b. Osteoid formation was evident at week 1, whereas mature, mineralized bone was observed by week 4. Quantification of bone area (%) revealed a slightly higher percentage for the hGFs‐MVs/TNP group at all time points; however, there was no significant difference at both 1‐ and 4‐week post‐implantation (Figure 5c). Regarding BIC %, there was no difference between groups initially at 1‐week post‐implantation. However, at 4 weeks, the hGFs‐MVs/TNP group showed significantly higher BIC % compared to the TNP group (Figure 5c).

### 3.4 Peri‐Implant Soft Tissue Formation Analysis

**FIGURE 6:**
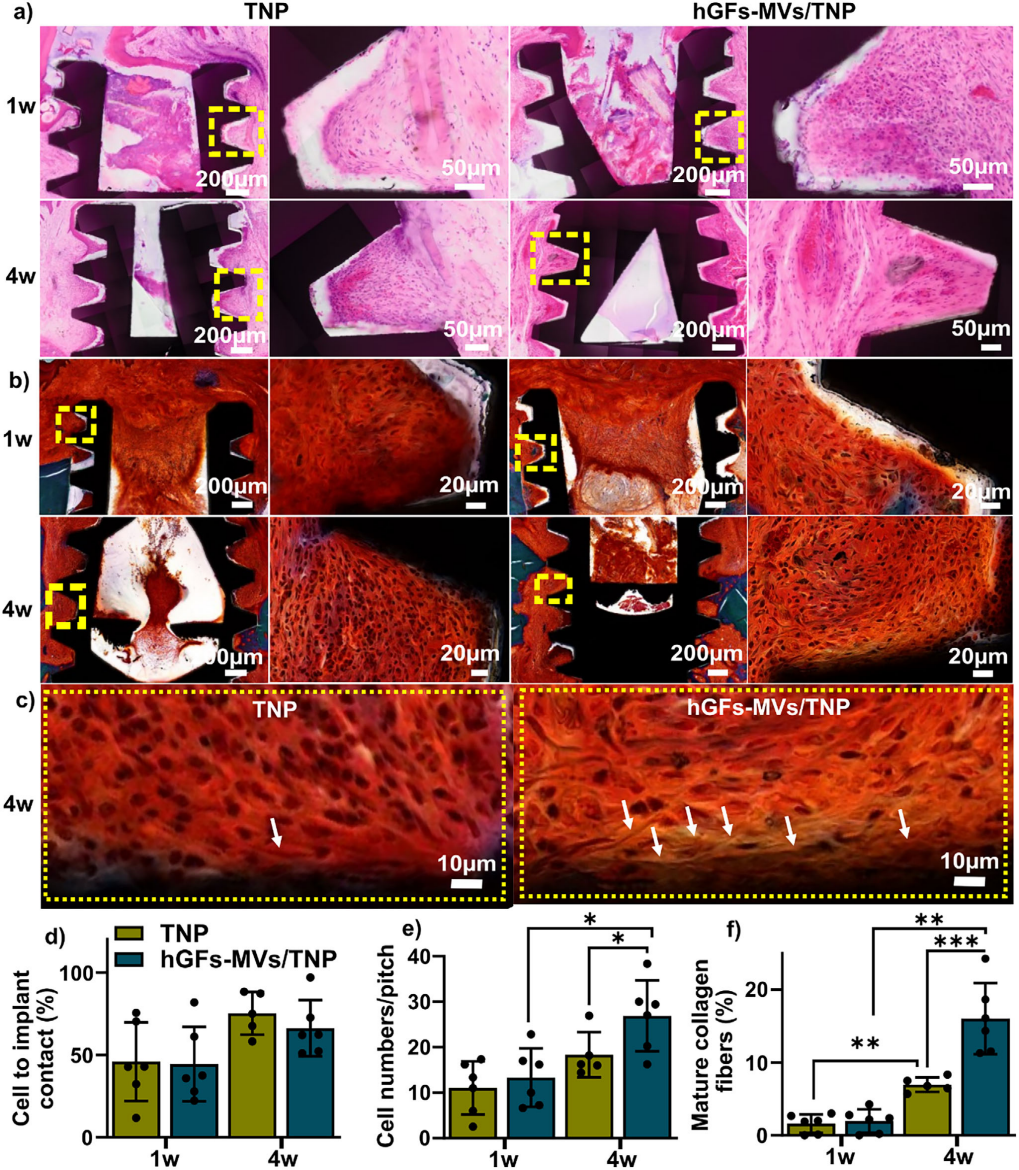
Peri‐implant soft tissue formation analysis. (a) H&E and (b) Golden trichrome staining of representative histological sections showing peri‐implant soft connective tissue formation within the implant pitch for TNP and hGFs‐MVs/TNP implants at 1‐ and 4‐weeks post‐implantation. The right panels in (a) and (b) show higher‐magnification views of the yellow‐boxed regions in the corresponding left panels. (c) Higher‐magnification view of collagen formation in the yellow‐boxed region in (b) at 4 weeks for both groups. White arrows indicate mature collagen fibers attached to the implant surface. (d) Quantification of soft connective tissue cell‐to‐implant contact (%; for first attached layer cells), connective tissue cell counts (for first attached layer cells) per pitch, and mature collagen fiber alignment (%)—excluding the implant threads and pitches with epithelial ingrowth‐ at 1 and 4 weeks for the TNP and hGFs‐MVs/TNP groups (n = 5–6). All data were analyzed using a two‐tailed unpaired *t*‐test. ^*^ *p*<0.05, ^**^, *p*<0.002, ^***^ *p*<0.002 between groups.

Peri‐implant soft tissue integration was assessed to evaluate the interface between the implant surface and surrounding soft tissues using histological images stained with H&E (Figure 6a) and Golden trichrome (Figure 6b), showing implant threads above the bone level. Both implants showed no difference in connective cell‐to‐implant contact (%) (Figure 6c). At 4 weeks post‐implantation, hGFs‐MVs/TNP implants exhibited significantly higher connective tissue cell attachment to implant surfaces compared to both their own levels at 1‐week post‐implantation and to the TNP group at the same time point (Figure 6d), indicating enhanced connective tissue cell adhesion over time with hGFs‐MVs treatment. Furthermore, the percentage of mature collagen fibers, which are thicker, more organized, and indicative of advanced peri‐implant soft tissue remodeling, increased significantly from week 1–4 in both groups (Figure 6e), consistent with normal healing. Notably, the hGFs‐MVs/TNP implants showed significantly greater levels of mature collagen fibers at week 4 compared to the TNP group (Figure 6e), suggesting that hGF‐MVs may accelerate peri‐implant soft tissue remodeling and enhance collagen maturation.

## 4 Discussion

Recent advances in implant surface engineering have demonstrated the significant potential of combining TNP implants with EVs, such as exosomes, to enhance osseointegration [5]. A growing body of in vitro and in vivo studies has investigated the synergistic effects of nano‐engineered Ti surfaces and EVs derived from MSCs or macrophages, showing enhanced osteogenic differentiation, mineralization, and modulation of immune responses [2, 10, 20, 22, 32, 33, 34, 35, 36, 37, 38, 39, 40]. Whilst the focal point of these studies was aimed at osseointegration outcomes, the equally critical contribution of peri‐implant soft tissue healing must also be acknowledged for long‐term implant stability, infection prevention, and functional restoration of the implant–tissue interface [2]. This study addresses this gap by functionalizing TNP implant surfaces with hGFs‐MVs to evaluate their effect on soft and hard tissue integration in vivo. At 4 weeks post‐implantation, the hGFs‐MV/TNP group showed significantly higher bone‐to‐implant contact (BIC %), increased soft tissue cell attachment, and a greater proportion of mature collagen fibers compared to the TNP‐only group. These results highlight the dual role of hGFs‐MVs in enhancing both bone formation and the integrity of the soft tissue seal, which is critical for long‐term implant stability and function.

Cells secrete a diverse range of EVs subtypes, ranging in size from 30 to 1000, including exosomes, MVs, and apoptotic bodies, each with distinct origins and functions [16, 41]. However, the overlapping size and shared EV surface markers (CD9, CD81, and CD63) among EV subtypes pose challenges to enrichment and downstream functional assays [29, 42]. MVs are typically formed by plasma membrane blebbing and enriched using differential centrifugation below 20 000 g after a low‐speed centrifuge (<300 g) to remove cells and debris [41, 43]. In our study, hGFs‐derived MVs were isolated at 16 000 g, yielding vesicles from 90–600 nm with a mode of ∼130 nm, consistent with MSC‐MVs isolated at the same speed (120–135 nm) [44]. In contrast, OB‐MVs and RBC‐MVs showed peak sizes at 450 nm and 81.2 nm [45] and ∼90 nm [46], respectively, likely due to differences in cell source and centrifugation speed (40 000 and 10 000 g). These size variations are likely due to differences in both cell source and centrifugation speed–16 000 g in our study and [44], compared to 40 000 g in [45] and 10 000 g in [46]. It should be noted that different centrifugation methods may generate a mixture of large EVs (MVs) and small EVs (exosomes), and it is not possible to isolate a pure MV population because no common MV‐specific surface markers are available. Such variability highlights the need for standardized MVs isolation protocols to ensure reproducibility and facilitate reliable interpretation of their bioactivity in tissue engineering applications.

Recent in vivo studies show that nanoengineered titanium implant surfaces alone or combined with exosomes from various cell sources enhance osseointegration by modulating osteoprogenitor and immune cell behavior [6]. Consistent with published reports on exosome‐ and microvesicle‐loaded TiO_2_ nanotube/nanopore systems, the surface roughness of the TNP implants remained unchanged following MV loading (Figure 3e), in agreement with previous studies showing that EV loading on TiO_2_ nanotubes does not alter implant surface nanotopography [25, 34, 40]. For instance, anodic oxidation‐treated TNTs enhanced BMSC‐exosome secretion and tibial bone‐implant contact [32], while also promoting osteogenesis and inhibiting osteoclastogenesis via the integrin β1/FAK/MAPK pathway in a tibial defect model [10]. In line with these reports, our study demonstrated a significant increase in bone–implant contact and peri‐implant bone formation following implantation of hGFs‐MVs‐modified titanium surfaces, supporting a pro‐osteogenic effect, although the specific signaling pathways were not directly examined. Notably, these results aligned with previous studies demonstrating enhanced bone formation using EVs derived from MSCs or macrophages on titanium surfaces [20, 21, 22]. For example, in diabetic conditions, M2‐EVs‐modified polydopamine‐coated Ti implants (PDA‐Ti) enhanced in vivo osseointegration via immunomodulation [22], with exosomes from TNF‐α‐treated hMSCs promoting osseointegration in distal femur defects of T2D mice by inducing M2 macrophage polarization through PI3K/AKT/mTOR pathway inhibition [20]. Consistent with these findings, our histomorphometric analysis revealed improved osseointegration in hGFs‐MVs‐treated implants; however, the involvement of macrophage polarization or specific immunomodulatory pathways was inferred based on literature rather than directly validated in the present study. Similarly, titania nanotubes (40 V, 95 nm) combined with M0‐exosomes in Matrigel promoted angiogenesis in vivo through the miR‐3473e/Akt1 pathway [37]. Exosomes from osteogenically differentiated hDPSCs loaded onto anodized Ti implants significantly enhanced BIC % in skull defects over 10 weeks, driven by osteogenic miRNAs (hsa‐miR‐29c‐5p, hsa‐miR‐378a‐5p, hsa‐miR‐10b‐5p, hsa‐miR‐9‐3p) [21]. While angiogenic signaling and miRNA profiles were not directly assessed, the enhanced peri‐implant bone formation observed in our model may be partly attributable to improved vascularization, a hypothesis supported by prior studies. Taken together, these studies highlight the diverse mechanisms through which EVs can promote bone regeneration and implant osseointegration. Our data further suggested that hGFs‐derived MVs exhibit osteogenic differentiation potential that might be comparable to exosomes from M0, M2 macrophages, and MSCs, highlighting their promise as a novel approach for improving implant osseointegration. While the present study focused on the in vivo performance of hGF‐MV‐functionalized TNP implants, previous in vitro studies suggest that MVs can modulate fibroblast behavior via ERK, JNK, and p38 MAPK signaling pathways [20, 37]. Gingival fibroblast‐ and epithelial‐derived MVs regulate inflammatory responses, extracellular matrix remodeling, and mineralization. These mechanisms may contribute to the enhanced soft tissue integration observed in our study. Future studies will aim to directly investigate MV cargo‐mediated signaling events in vivo to fully elucidate the molecular basis of their bioactivity.

The role of soft tissue attachment in long‐term maintenance of peri‐implant health, and ultimately, the reduction of biologic complications, cannot be understated. The quality of the peri‐implant soft tissue seal is critical for creating a stable and infection‐resistant line of defense around the implant‐mucosal interface, minimizing bacterial invasion, reducing inflammation, and improving long‐term implant success [1, 2, 5]. Our data showed that hGFs‐MVs/TNP not only enhanced bone‐to‐implant integration but also improved soft tissue healing through increasing soft tissue cell attachment and promoting mature collagen fiber orientation, compared to the TNP‐only control. These are important features for promoting the soft tissue seal around the transmucosal implant collar. This accelerated collagen maturation may enhance the mechanical stability of the peri‐implant tissue and improve soft tissue healing around the implant. Enhanced collagen organization is likely to result in more robust connective tissue attachment within the soft tissue component at the implant site, potentially lowering the risk of microbial infiltration and resultant peri‐implant inflammation, thus supporting long‐term implant integration [47]. This phenomenon was consistent with previously published in vitro findings demonstrating the function of hGFs‐MVs in modulating inflammatory responses in epithelial cells [25] and adipogenesis in MSCs [19] alongside the synergistic potential of TNP themselves to promote ligamentous differentiation in hGFs [11]. Collectively, these results underpin the utility of human gingival fibroblast‐derived MVs as a novel strategy that promotes early bone formation and contributes to enhanced soft tissue healing, offering a dual benefit.

Nevertheless, it is important to consider that EV loading amounts and cell sources vary significantly across studies. Our study used 10^9^ hGFs‐MVs per 2 mm TNP implant at room temperature for 1 h; M2‐EVs (100 µg/mL) were loaded onto PDA‐Ti overnight at 4°C [22]; in contrast,150 µg of exosomes were incubated at 4°C for 12 h with a 4 mm anodized Ti implant [21] and TNF‐MSCs‐exosome 10^11^/cm^2^ in 1 mm acid‐etching Ti rods at 4°C overnight [20]. Such differences in EV dosage and loading protocols may affect the functional in vivo outcomes observed in these studies. Furthermore, exosomes derived from different cell sources likely contain distinct compositions that contribute variably to the EV cargo. For instance, MSCs‐exosomes are enriched with cell adhesion molecules and signaling proteins abundant in the exosome proteome after immobilization to Ti discs [48]. Meanwhile, M2‐EVs carry miR‐23a‐3p, which inhibits NOD‐like receptor protein 3 (NLRP3) inflammasome activation in M1 macrophages and reduces inflammatory cytokines such as IL‐1β by targeting NEK7 [22]. Although we observed favorable bone–implant integration in the EV‐treated group, immune cell infiltration and cytokine expression were not directly assessed in our rat model, as resin‐embedded hard tissue sections are not readily amenable to immunohistochemical identification of immune cell populations. Another advantage of using extracellular vesicles, compared to cell‐based therapies, is their low immunogenicity, even in cross‐species (xenogeneic) contexts. In preclinical studies, human EVs (e.g., from HEK293T or MSCs) caused minimal immune activation in rodent models, with negligible toxicity and reduced immune response compared with parent cells [49, 50]. Nevertheless, we did not directly assess immune cell infiltration or cytokine induction in our rat model, as resin‐embedded hard tissue sections are not readily amenable to immunohistochemistry for identifying immune cell types. These observations highlight the importance of choosing appropriate EV sources tailored to specific implant applications and desired therapeutic outcomes, while acknowledging that the molecular mechanisms discussed here are inferred through integration of our findings with established literature rather than directly demonstrated within the scope of this study.

This study focused on early healing over a short 4‐week in vivo period, which may not fully capture late soft tissue maturation, bone remodeling changes, and long‐term implant stability. Resin‐embedded hard tissue sections prevented direct assessment of immunochemistry, immune cell infiltration, cytokine expression, or molecular mechanisms underlying hGFs‐MVs effects. The integrity of the soft tissue seal to the fully functional dental prosthesis cannot be concluded from these preliminary proof‐of‐concept results. Additionally, EVs from other cell sources were not evaluated, and it remains unclear whether the observed improvements are specific to hGFs‐MVs. Furthermore, the discussion of potential molecular mechanisms relies on previously published studies and should be interpreted as hypothesis‐generating rather than directly supported by the present data. Future research should therefore focus on understanding long‐term tissue stability, functional outcomes, and including alternative EV sources to clarify cell‐specific effects under clinically relevant function. Despite limitations, our findings highlight the promising role of hGFs‐derived MVs in improving both peri‐implant bone and soft tissue integration, offering new opportunities to improve patient outcomes in dental and orthopedic applications.

## 5 Conclusions

This ‘proof of concept’ in vivo study shows that hGFs‐MVs combined with TNPs significantly improve bone and soft tissue integration around titanium implants in the oral cavity. By enhancing both osseointegration and soft tissue healing, this dual approach offers promising potential to improve implant treatment success. Further research is needed to confirm the long‐term stability of the peri‐implant issues over time and clarify underlying biological mechanisms.

## Conflicts of Interest

The authors declare no conflicts of interest.

## Data Availability

The data that support the findings of this study are available from the corresponding author upon reasonable request.
